# Crowded

**Published:** 1878-02-20

**Authors:** 


					﻿EDITORIAL AND MISCELLANEOUS.
CROWDED.
It is a general remark that t he professions are
crowded. This we know to be the case with ours—
the time-honored medical profession. This condi-
tion of things is not a subject of congratulation. It
lowers the dignity of the calling. It brings hard-
ships and trials to many good men who have missed
other opportunities by missing their vocations
Of the legion of young men who are annually em-
barking in medieal study, but few are mentally
fitted for the undertaking. Not because of a want
of education, perseverance or energy, but because
they blindly seek to enter a profession for the du
ties and responsibilities of which they are unfitted
by reason of inherent unadaptability. Nature never
mistakes. She never makes a doctor out of a
farmer or a mechanic, and when young men fly in
her face, endeavoring to turn her from her course
in this special case, they surely and inevitably
fail—fail in the profession, and fail in acquiring a
support for themselves and their families.
We would discourage young men from entering
the professions. They are bo crowded that even
there is no room at the top. They are so crowded
that the effort to exist subjects them to a pressure
of temptation to do wrong, almost resistless.
Quackery and quacks abound on every side from
this cause. A living must be had, if not by honest
means, then at all hazards. For this reason the
ethics of the profession has lost its power and is
trampled in the dust.
In the United States there is one physician for
every 600 inhabitants—a proportion greater, by
far, than any other country on the globe. We have
more isms, more colleges, more poor, perishing
doctors than any other country. We have more
quacks, more patent medicines and more general
misery in our ranks than in all Europe combined.
It is time for our young men to pause. Let them
think. Let our medical brethren help them to
think. Tell them the true state of the profession,
and implore them to give their time and talents to
other pursuits, and not wreck themselves upon the
rooks and quicksands of a “ profession ” which,
however beautifully pictured, (as a mirage) in the
future,will prove a delusion,and ruinous in the end .
Save them from the temptation to launch them-
selves on a sea of quackery, and thus to dishonor a
calling so illustrious. Save them from want, the
cry of starving families, and the sight of misery and
rags at home. Save them, that the country, no
less than the profession, may be saved. Encourage
them to till the soil, to educate the heart, to dig-
nify a noble manhood and follow Providence—
Providence, who has no need of a poor doctor, a
poor pretender in any calling in life.
				

